# Predictors of mortality from extended-spectrum beta-lactamase-producing Enterobacteriaceae bacteremia

**DOI:** 10.1080/22221751.2023.2217951

**Published:** 2023-06-05

**Authors:** Hiroki Namikawa, Waki Imoto, Koichi Yamada, Yoshihiro Tochino, Yukihiro Kaneko, Hiroshi Kakeya, Taichi Shuto

**Affiliations:** a Department of Medical Education and General Practice, Graduate School of Medicine, Osaka Metropolitan University, Osaka, Japan; b Department of Infection Control Science, Graduate School of Medicine, Osaka Metropolitan University, Osaka, Japan; c Research Center for Infectious Disease Sciences, Graduate School of Medicine, Osaka Metropolitan University, Osaka, Japan; d Department of Bacteriology, Graduate School of Medicine, Osaka Metropolitan University, Osaka, Japan

**Keywords:** Extended-spectrum beta-lactamase, Enterobacteriaceae, bacteremia, mortality, meta-analysis

## Abstract

Extended-spectrum beta-lactamase-producing Enterobacteriaceae (ESBL-PE) bacteremia can have poor clinical outcomes. Thus, determining the predictors of mortality from ESBL-PE bacteremia is very important. The present systematic review and meta-analysis aimed to evaluate studies to determine predictors associated with ESBL-PE bacteremia mortality. We searched PubMed and Cochrane Library databases for all relevant publications from January 2000 to August 2022. The outcome measure was mortality rate. In this systematic review of 22 observational studies, 4607 patients with ESBL-PE bacteremia were evaluated, of whom 976 (21.2%) died. The meta-analysis showed that prior antimicrobial therapy (RR, 2.89; 95% CI, 1.22–6.85), neutropenia (RR, 5.58; 95% CI, 2.03–15.35), nosocomial infection (RR, 2.46; 95% CI, 1.22–4.95), rapidly fatal underlying disease (RR, 4.21; 95% CI, 2.19–8.08), respiratory tract infection (RR, 2.12; 95% CI, 1.33–3.36), Pitt bacteremia score (PBS) (per1) (RR, 1.35; 95% CI, 1.18–1.53), PBS ≥ 4 (RR, 4.02; 95% CI, 2.77–5.85), severe sepsis (RR, 11.74; 95% CI, 4.68–29.43), and severe sepsis or septic shock (RR, 4.19; 95% CI, 2.83–6.18) were found to be mortality predictors. Moreover, urinary tract infection (RR, 0.15; 95% CI, 0.04–0.57) and appropriate empirical therapy (RR, 0.39; 95% CI, 0.18–0.82) were found to be a protective factor against mortality. Patients with ESBL-PE bacteremia who have the aforementioned require prudent management for improved outcomes. This research will lead to better management and improvement of clinical outcomes of patients with bacteremia caused by ESBL-PE.

## Introduction

Extended-spectrum beta-lactamases (ESBLs) are enzymes that confer resistance to various types of beta-lactam antibiotics, including oxyimino-cephalosporins (cefotaxime, ceftriaxone, cefuroxime, cefixime, ceftazidime, cefepime, and cefpirome) and monobactams (aztreonam) [1, 2]. ESBLs are produced by Enterobacteriaceae bacteria, mainly *Escherichia coli* and *Klebsiella pneumoniae* [3]. In recent years, the spread of ESBL-producing Enterobacteriaceae (ESBL-PE) has increased rapidly worldwide [4]. Patients with ESBL-PE bacteremia can have poor clinical outcomes due to delayed appropriate antimicrobial therapy and limited therapeutic options [5]; hence, ESBL-PE has become a clinically critical issue. Recent studies reported mortality rates ranging from approximately 12% to 41% among patients with ESBL-PE bacteremia [6-11]. Therefore, when treating patients with ESBL-PE bacteremia, determining the predictors of mortality from ESBL-PE bacteremia is very important.

Several recent studies have reported different risk factors associated with ESBL-PE bacteremia mortality, including nosocomial infection [10, 11], transfer to intensive care unit [12, 13], respiratory tract infection [6, 12], non-urinary tract infection [13, 14], age [10, 14], and severe sepsis or septic shock [8, 9, 11, 14]. However, our literature search revealed no study focusing on the meta-analysis of predictors associated with ESBL-PE bacteremia mortality. The present systematic review and meta-analysis aimed to evaluate studies in order to determine predictors associated with ESBL-PE bacteremia mortality.

## Materials and methods

### Literature review

The systematic review and meta-analysis were performed following the Preferred Reporting Items for Systematic Reviews and Meta-Analyses (PRISMA) guidelines. Furthermore, the research model was constructed based on previous studies [15,16], with slight modifications. A methodical search for the reviewed literature was conducted in PubMed and Cochrane Library databases for all publications from January 2000 to August 2022. Our search comprised three keywords or phrases: “extended spectrum beta lactamase or ESBL,” “bacteremia or bloodstream infection,” and “mortality or fatality or lethality or prognosis or predictor.” The search was conducted taking into account all the three keywords and the phrases in combination. Results were restricted to full-text articles available in English.

For the purpose of our review, we included clinical trials, cohort studies, case–control studies, and cross-sectional studies that had determined cases of ESBL-producing Enterobacteriaceae bacteremia. By contrast, reviews, systematic reviews, meta-analyses, guidelines, editorials, letters to the editor, comments, case reports, animal research, in vitro studies, research focused on children, studies involving < 20 patients per group, and studies performing inappropriate multivariate analysis using automatic selection methods such as stepwise regression and wherein only items with a predefined small *p*-value in the univariate analysis were included in the multivariate regression model were excluded. Both monomicrobial and polymicrobial episodes were considered for inclusion. The primary outcome was mortality. We did not have any limitation in place regarding the cause of death (infection-attributed or not) or the days to death.

### Data extraction and quality assessment

Data were extracted from the literature by two independent reviewers and standardized using an established format that included the following study variables: first author, country, study design, year of publication, study duration, number of patients with ESBL-producing Enterobacteriaceae bacteremia, mortality, and statistical analysis method. Discrepancies or disagreements, if any, were resolved by mutual discussion and consensus. We performed quality assessment using the Newcastle–Ottawa Scale.

### Statistical analyses

Multivariate model-adjusted measurements were used as main effect estimates. Risk ratio (RR; odds ratio or hazard ratio [HR]) is an appropriate effect estimate for cohort and case–control studies, and only studies that reported or allowed for the calculation of RR and error estimates (confidence intervals [CIs] and standard error) were included in the quantitative data synthesis. We performed a meta-analysis of the parameters for which RR was reported in three or more studies and wherein at least one statistically significant association was identified. Furthermore, we conducted a subgroup analysis to compare the clinical effectiveness of carbapenems and carbapenem-sparing regimens. We estimated the RR and 95% CIs for all-cause mortality based on the number of individuals at risk and number of deaths. We performed a meta-analysis of identical regimens in three or more studies. As the background factors and combinations of confounding factors were different in different articles, a meta-analysis was conducted using a random-effects model. The results of the meta-analysis are presented as forest plots; the Cochran's Q test was used to assess heterogeneity. An *I*^2^ value between 50% and 100% was a prerequisite for considering the presence of statistical heterogeneity. Publication bias and small-study effects were investigated by visually assessing the funnel plots. All statistical analyses were performed with EZR (Saitama Medical Center, Jichi Medical University, Saitama, Japan), which is a modified version of the R Commander that includes the statistical functions that are frequently used in biostatistics.

## Results

### Study selection and characteristics

**Figure 1.**
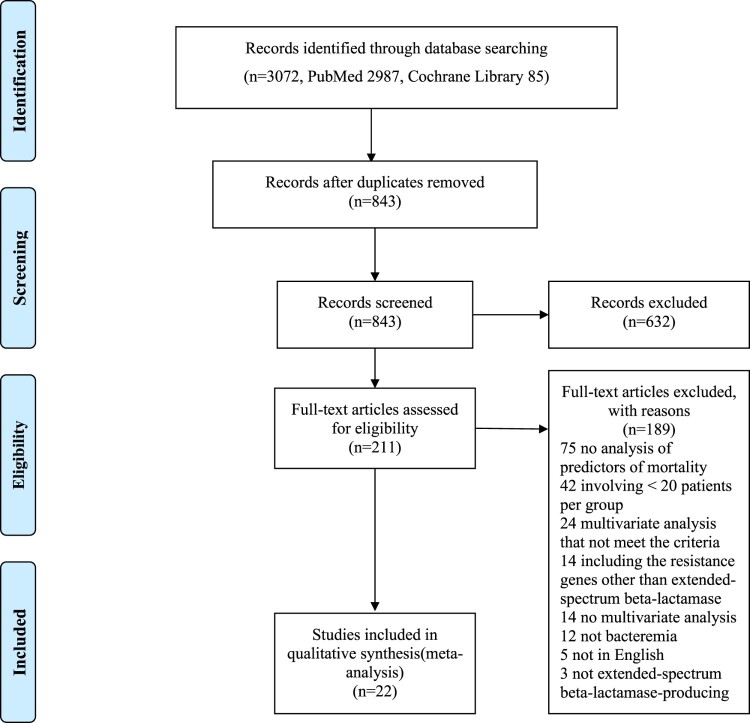
Flow diagram of selection process for included studies.

Figure 1 summarizes the study identification process in the form of a PRISMA flow diagram. The search revealed that 3072 existent studies were relevant to the present literature review. After the elimination of 2229 duplicates, 843 records were screened based on the title and abstract. Of these, 632 records were further eliminated after we reviewed the title and/or the abstract. The remaining 211 papers were assessed in a full-text review, and 189 articles were further excluded at this stage for the following reasons: no analysis of the predictors of mortality (n = 75), did not meet the case-load criteria (n = 42), multivariate analysis that did not meet the required criteria (n = 24), inclusion of resistance genes other than ESBL (n = 14), no multivariate analysis (n = 14), no bacteremia (n = 12), not in English (n = 5), and not including ESBL-PE (n = 3) (Figure 1). After a complete textual appraisal, 22 studies were included in the present review [9-11,13,17-34]. The key attributes of the selected articles are summarized in Table 1. These studies were published between 2004 and 2022. The studies were carried out in 16 countries (Korea, Italy, Spain, Taiwan, the United States of America, Israel, Singapore, Germany, Greece, Turkey, South Africa, Canada, Argentina, France, China, and Japan) on five continents (Asia, Europe, North America, Africa, and South America) and were all observational; one was a prospective study, 20 were retrospective studies, and the study type of one was unknown. The sample size ranged from 48 to 622. A total of 4607 patients with ESBL-PE bacteremia were evaluated, of whom 976 (21.2%) died. The assessment of the outcomes was set at 30 days in more than half of the studies.

**Table 1.** Characteristics of studies included in the systematic literature review and meta-analysis.

Author (year)	Country	Design	Period	Population	Death (%)	Mortality day
Kang et al. [17]	Korea	Retrospective cohort study	1998–2002	133 patients with ESBL-producing *E. coli* or *K. pneumoniae* bacteremia	34 (25.6)	30
Tumbarello et al. [22]	Italy	Retrospective cohort study	1999–2005	129 patients with ESBL-producing *E. coli* bacteremia	38 (29.5)	21
Rodríguez-Baño et al. [23]	Spain	Prospective study	2004–2006	96 patients with ESBL-producing *E. coli* bacteremia	24 (25.0)	14
Wang et al. [24]	Taiwan	Retrospective cohort study	2002–2007	113 patients with ESBL-producing *E. coli* or *K. pneumoniae* bacteremia	27 (23.9)	14
Chung et al. [25]	Taiwan	Observational study	2005–2010	124 patients with ESBL-producing *E. coli* bacteremia	30 (24.2)	28
Lee et al. [26]	Taiwan	Retrospective study	2002–2007	251 patients with ESBL-producing *E. coli* or *K. pneumoniae* bacteremia	33 (13.1)	sepsis-related
Ku et al. [21]	Korea	Retrospective cohort study	2006–2010	191 patients with ESBL-producing *E. coli* or *K. pneumoniae* bacteremia	47 (24.6)	28
Tamma et al. [27]	USA	Retrospective cohort study	2007-2014	213 patients with ESBL-PE bacteremia	26 (12.2)	14
Ofer-Friedman et al. [28]	Israel and USA	Retrospective cohort study	2008–2012	79 patients with ESBL-PE bacteremia	39 (49.4)	90
Lee et al. [29]	Taiwan	Retrospective case-control study	2007–2012	389 patients with ESBL-producing *E. coli* or *K. pneumoniae* bacteremia	71 (18.3)	30
Cheng et al. [20]	Taiwan	Retrospective study	2002–2010	111 patients with bacteremic pneumonia caused by ESBL-producing *E. coli* or *K. pneumoniae*	45 (40.5)	30
Ng et al. [30]	Singapore	Retrospective cohort study	2011–2013	151 patients with ESBL-producing *E. coli* or *K. pneumoniae* bacteremia	46 (30.5	30
Palacios-Baena et al. [18]	Multinational	Retrospective cohort study	2004–2013	622 patients with ESBL-PE bacteremia	115 (18.5)	30
Yu et al. [31]	Taiwan	Retrospective study	2009–2010	48 patients with ESBL-producing *K. pneumoniae* bacteremia	27 (56.3)	in hospital
Lo et al. [32]	Taiwan	Retrospective cohort study	2008–2010	299 patients with ESBL-producing *E. coli* or *K. pneumoniae* bacteremia	66 (22.1)	30
Chapelet et al. [33]	France	Retrospective cohort study	2008–2015	140 patients with ESBL-producing *E. coli* bacteremia	22 (15.7)	30
Ko et al. [13]	Korea	Retrospective cohort study	2010–2014	232 patients with ESBL-PE bacteremia	23 (9.9)	30
Xiao et al. [19]	China	Retrospective study	2013-2016	283 patients with ESBL-producing *E. coli* bacteremia	42 (14.8)	28
Zohar et al. [9]	Israel	Retrospective cohort study	2014-2017	193 patients with ESBL-PE bacteremia	32 (16.6)	30
Mitsuboshi et al. [34]	Japan	Retrospective cohort study	2012-2016	179 patients with ESBL-PE bacteremia	24 (13.4)	30
Benetazzo et al. [10]	France	Retrospective cohort study	2011-2018	307 patients with ESBL-PE bacteremia	125 (40.7)	30
Park et al. [11]	Korea	Retrospective cohort study	2013-2020	324 patients with ESBL-PE bacteremia	40 (12.3)	30

Notes: *E. coli*: *Escherichia coli*, ESBL-PE: extended-spectrum β-lactamase-producing Enterobacteriaceae, *K. pneumoniae*: *Klebsiella pneumoniae*. Multinational: Spain, Germany, Italy, Greece, Israel, Turkey, South Africa, Canada, USA, Argentina, and Taiwan, USA: United States of America.

### Meta-analysis

Table 2 lists the parameters for which the RR for death caused by ESBL-PE bacteremia was reported in the multivariate analysis of the 22 selected studies. The parameters for which the RR was reported in three or more studies and for which at least one statistically significant association was identified were prior antimicrobial therapy (within 30 days before bacteremia), neutropenia, nosocomial infection, Charlson score, rapidly fatal underlying disease, respiratory tract infection (especially pneumonia), urinary tract infection, Pitt bacteremia score (PBS; per1), PBS ≥ 4, severe sepsis, severe sepsis or septic shock, appropriate empirical therapy, and piperacillin/tazobactam.

**Table 2.** Results of studies performing multivariable analyses regarding mortality in patients with ESBL-PE bacteremia.

Author (year)	N	Methods	Predictor	RR	95% CI	*P*-value
Kang et al. [17]	133	Logistic regression	Administration of broad-spectrum	9.18	1.55–54.51	0.015
			cephalosporin as definitive antimicrobial			
			therapy (/No)			
			Neutropenia (/No)	9.03	1.24–65.97	0.030
			Peritonitis (/No)	10.25	1.26–83.25	0.029
			Presentation with septic shock (/No)	45.25	6.55–312.84	<0.001
			Increasing APACHE II score (per 1)	1.44	1.11–1.87	0.006
Tumbarello et al. [22]	129	Logistic regression	Inadequate initial antimicrobial treatment (/No)	6.22	2.33–16.61	<0.001
			Unknown source (/No)	4.28	1.71–10.69	0.001
			Presentation with septic shock (/No)	5.88	1.26–27.45	0.02
Rodríguez-Baño et al. [23]	96	Conditional	Pitt score >1 (/0-1)	3.9	1.2–12.9	0.02
		logistic regression	High-risk source^a^ (/No)	5.5	1.4–21.9	0.01
			Severe sepsis or shock (/No)	4.6	1.4–15.2	0.01
			Resistance score >3 (/0-3)	6.5	1.4–30.0	0.01
Wang et al. [24]	113	Logistic regression	Severe sepsis (/No)	24.29	5.62–104.98	<0.001
			Pneumonia (/No)	5.20	1.29–20.95	0.021
			Respiratory failure (/No)	3.45	0.40–29.89	0.261
			Shock (/No)	2.81	0.43–18.42	0.280
			Pitt bacteremia score ≥4 (/< 4)	2.63	0.47–14.64	0.269
			Appropriate empirical therapy (/No)	0.345	0.06–2.03	0.239
			Appropriate definitive therapy (/No)	0.089	0.01–0.58	0.011
Chung et al. [25]	124	Logistic regression	Cancer (/No)	2.81	1.03–7.66	-
			Community-onset (/No)	0.29	0.11–0.77	-
			Shock (/No)	6.75	2.52–18.0	-
Lee et al. [26]	251	Conditional	Age (per 1-yr)	1.01	0.98–1.04	0.42
		logistic regression	Male (/female)	1.13	0.45–2.86	0.80
			Severe sepsis (/No)	15.9	5.84–43.34	<0.001
			Hospital-onset bacteremia (/No)	4.65	1.42–15.24	0.01
			Rapidly fatal underlying disease (/No)	2.07	0.7–6.17	0.20
			Pneumonia (/No)	1.56	0.63–3.88	0.34
			Appropriate antimicrobial therapy (/No)	0.44	0.14–2.36	0.57
			Ertapenem-nonsusceptible isolates^b^ (/No)	5.12	2.04–12.88	0.001
Ku et al. [21]	191	Logistic regression	Age (per 1-yr)	1.066	0.976–1.165	0.157
			Hospital-acquired (/community-acquired)	0.292	0.067–1.281	0.103
			Hemodialysis (/No)	1.205	0.257–5.658	0.813
			Neutropenia at bacteremia (/No)	3.592	0.955–13.505	0.058
			Use of steroids at bacteremia (/No)	1.188	0.384–3.673	0.765
			Prior antimicrobial therapy within 30 days	9.084	1.570–52.572	0.014
			before bacteremia (/No)			
			Urinary tract (/No)	0.076	0.010–0.547	0.011
			Pulmonary (/No)	0.948	0.328–2.743	0.922
			SOFA score (per 1)	1.847	1.493–2.286	<0.001
Tamma et al. [27]	213	Adjusted	Piperacillin-tazobactam (/carbapenem)	1.92	1.07–3.45	0.03
		cox regression	Age (per 10-yr increase)	1.18	0.99–1.41	0.07
			Pitt bacteremia score (per 1)	1.49	1.28–1.72	<0.001
			ICU level care, day 1 (per 1)	4.25	1.86–9.71	<0.001
Ofer-Friedman et al. [28]	79	Logistic regression	Piperacillin-tazobactam case (/carbapenem)	7.9	1.2–53	0.03
			Time at risk^c^ (per 1 d)	1.1	1.008–1.13	0.03
			Fatal McCabe score (per 1)	26	6–115	<0.001
Lee et al. [29]	389	Logistic regression	Pitt bacteremia score ≥4 (/< 4)	3.2	1.5–6.6	<0.01
			Pneumonia (/No)	4.9	2.5–9.9	<0.01
			Urosepsis (/No)	0.3	0.1–0.8	0.02
			Definitive flomoxef therapy (/carbapenem)	1.4	0.5–4.2	0.52
			Definitive flomoxef therapy for isolates with	5.7	1.9–16.8	<0.01
			flomoxef MICs of 2-8 mg/L (/No)			
Cheng et al. [20]	111	Conditional	Solid tumour (/No)	2.09	0.53–8.29	0.30
		logistic regression	Rapidly fatal underlying disease (/No)	5.75	1.54- 21.48	0.009
			Critical illness (Pittsburg bacteremia score of ≥4) (/< 4)	4.28	1.35–13.57	0.013
			Severe sepsis (/No)	4.84	1.55–15.14	0.007
			Appropriate empirical antimicrobial therapy	0.19	0.77–0.55	0.002
			(/No)			
Ng et al. [30]	151	Logistic regression	Pitt bacteremia score (per 1)	1.20	0.98–1.48	0.08
			Charlson's comorbidity index (per 1)	0.94	0.81–1.09	0.40
			Respiratory source (/No)	2.81	0.87–9.05	0.08
			Hepatobiliary source (/No)	0.18	0.02–1.48	0.11
			Unknown source (/No)	1.51	0.33–6.92	0.60
			Empiric piperacillin-tazobactam (/carbapenem)	0.99	0.45–2.17	0.99
Palacios-Baena et al. [18]	622	Logistic regression	Age >50 years (/≤50)	2.63	1.18–5.85	0.01
			*Klebsiella* spp. (/No)	2.08	1.21–3.58	0.008
			Source other than UTI (/No)	3.60	2.02–6.44	<0.001
			McCabe (UF and RF) (/No)	3.91	2.24–6.80	<0.001
			Pitt score >3 (/≤3)	3.04	1.69–5.47	<0.001
			Severe sepsis/septic shock (/No)	4.80	2.72–8.46	<0.001
			Inappropriate early targeted therapy (/No)	2.47	1.58–4.63	0.002
Yu et al. [31]	48	Cox proportional	Nosocomial (/No)	2.29	0.41–12.80	-
		hazards regression	Urinary tract infection (/primary infection)	0.92	0.15–5.64	-
			Stay in intensive care unit (/No)	0.99	0.22–4.45	-
			No removal of CVC (/no CVC line)	0.83	0.21–3.21	-
			Initial appropriate antibiotic therapy (/No)	0.88	0.28–2.77	-
			Charlson score (per 1)	1.43	1.04–1.99	<0.05
			APACHE II score ≥15 (/< 15)	2.55	0.82–7.88	-
Lo et al. [32]	299	Conditioning	Hospital-onset bacteremia (/No)	2.57	1.22–5.45	0.01
		logistic regression	Pneumonia (/No)	1.27	0.65–2.48	0.49
			Urosepsis (/No)	0.47	0.18–1.18	0.12
			Rapidly fatal underlying disease (/No)	5.73	2.51–13.08	<0.001
			Pitt bacteremia score ≥4 points (/< 4)	7.09	3.71–13.56	<0.001
			Fluoroquinolone definitive therapy	0.18	0.03–0.92	0.04
			(/carbapenem)			
Chapelet et al. [33]	140	Logistic regression	Age (per 1)	1.01	0.96–1.07	0.784
			Female (/male)	0.85	0.18–4.09	0.836
			Charlson score comorbidities index ≥2 (/< 2)	5.00	0.28–88.36	0.273
			History of hepatic disease (/No)	0.47	0.05–4.79	0.522
			Dementia (/No)	54.51	1.20–2472.22	0.040
			Walking status (able to walk) (/No)	0.03	0.01–0.59	0.021
			Immunosuppressive treatment, including	1.58	0.12–21.55	0.733
			corticosteroid (/No)			
			Previous antimicrobial therapy within 30 days	3.34	0.64–17.50	0.153
			before bacteremia (/No)			
			Time to blood culture positivity ≤ 480 min	3.20	0.48–21.13	0.228
			(/>480 min)			
			SOFA score (per 1)	1.69	1.26–2.27	<0.001
			Neutropenia (/No)	12.94	1.01–166.00	0.049
			Appropriate empirical antibiotic-treatment	0.42	0.08–2.21	0.305
			(/No)			
			Urinary tract infection (/No)	0.07	0.01–0.84	0.036
			Pulmonary tract infection (/No)	1.17	0.06–24.61	0.921
Ko et al. [13]	232	Cox proportional	Empirical non-carbapenem use (/carbapenem)	0.83	0.24–2.82	0.76
		hazard regression	Age (per 1)	1.02	0.99–1.06	0.14
			*K. pneumoniae* infection (/*E. coli* infection)	2.73	0.83–9.00	0.10
			Antibiotic administration interval^d^ (per 1 h)	1.02	0.98–1.05	0.32
			Catheter-related BSI (/primary bacteremia)	2.38	0.34–16.77	0.38
			Urinary tract (/primary bacteremia)	0.07	0.01–0.51	0.01
			Intra-abdominal (/primary bacteremia)	1.30	0.41–4.13	0.66
			Others^e^ (/primary bacteremia)	0.61	0.09–4.17	0.61
			Transfer to ICU within 48 h (/No)	4.99	1.85–13.46	<0.01
			APACHE II (per 1)	1.03	0.96–1.09	0.45
			Charlson’s WIC (per 1)	0.99	0.84–1.17	0.93
Xiao et al. [19]	283	Binary logistic	Lung infection (/No)	2.012	0.912–4.437	0.083
		regression	Urinary catheterization (/No)	1.879	0.891–3.963	0.097
			Prior antibiotics use^f^ (/No)	1.872	0.886–3.954	0.100
			Total albumin (median, IQR)	0.941	0.887–0.999	0.045
			APACHEII score (per 1)	1.103	1.033–1.177	0.003
Zohar et al. [9]	193	Logistic regression	Age (per 1)	1.04	0.99–1.09	0.093
			Charlson comorbidity index (per 1)	1.21	1.01–1.46	0.040
			Other than *E. coli* (/*E. coli*)	1.78	1.06–3.01	0.031
			severe sepsis or septic shock (/No)	3.85	1.69–8.77	0.001
Mitsuboshi et al. [34]	179	Logistic regression	Age ≥ 85 years (/< 85 years)	1.59	0.36–7.02	0.54
			qSOFA scores ≥2 (/< 2)	1.27	0.27–5.97	0.76
			Biliary tract infection (/urinary tract infection)	8.90	0.88–89.90	0.06
			Other sites of infection (/urinary tract infection)	27.50	2.90–260.00	<0.01
Benetazzo et al. [10]	307	Cox proportional	Aminoglycoside (/No)	1.05	0.54–2.06	0.89
		hazard regression	Male sex (/female)	0.50	0.25–1.01	0.05
			55≦age<62 (/<55)	1.64	0.67–4.03	0.28
			62≦age<70 (/<55)	1.49	0.60–3.65	0.39
			Age ≥ 70 (/< 55)	2.67	1.09–6.54	0.03
			Medical admission (/No)	0.72	0.28–1.88	0.51
			Cardiac insufficiency (/No)	2.16	0.78–5.98	0.14
			Transplantation (/No)	5.20	1.4–19.35	0.01
			Hospital acquired infection (/No)	8.67	1.74–43.08	0.01
			5≦SOFA<7 (/<5)	0.54	0.21–1.42	0.76
			7≦SOFA<11 (/<5)	0.52	0.23–1.18	0.12
			SOFA ≥ 11 (/< 5)	1.69	0.66–4.34	0.28
			Duration of vasopressors			
			between 24 and 48 h (/< 24 h)	3.02	1.24–7.31	0.01
			>48 h (/< 24 h)	3.61	1.62–8.02	0.002
			Active combination therapy (/No)	0.55	0.28–1.08	0.08
			ARDS (/No)	2.42	1.14–5.16	0.02
			Acute renal failure	2.49	1.14–5.47	0.02
Park et al. [11]	324	Cox proportional	Male sex (/female)	0.91	0.43–1.91	0.80
		hazard regression	*E. coli* (/No)	0.38	0.17–0.83	0.015
			Nosocomial acquisition (/No)	2.68	1.32–5.42	0.006
			Liver cirrhosis (/No)	1.76	0.58–5.37	0.32
			ESRD (/No)	1.87	0.28–12.57	0.52
			Solid tumour, localized (/No)	1.96	0.93–4.11	0.077
			Metastatic solid tumour (/No)	3.96	1.28–12.24	0.017
			Chemotherapy within 6 months (/No)	1.27	0.47–3.44	0.64
			Charlson’s comorbidity index (per 1)	1.03	0.85–1.25	0.75
			Biliary (/urinary tract)	1.42	0.45–4.43	0.55
			Other (/urinary tract)	1.73	0.74–4.05	0.21
			Pitt bacteremia score (per 1)	1.29	1.06–1.56	0.012
			Severe sepsis or septic shock (/No)	3.14	1.30–7.59	0.011
			Ertapenem (/other carbapenem)	0.60	0.29–1.22	0.16

Notes: APACHE: acute physiology and chronic health evaluation, ARDS: acute respiratory distress syndrome, BSI: bloodstream infection, CI: confidence interval, CVC: central venous catheter, *E. coli*: *Escherichia coli*, ESRD: end-stage renal disease, ICU: intensive care unit, *K. pneumoniae*: *Klebsiella pneumoniae*, Method: methods of multivariate analysis, MIC: minimum inhibitory concentration, N: sample size, qSOFA: quick SOFA, RF: rapidly fatal, RR: risk ratio, SD: standard deviation, SOFA: sequential organ failure assessment, UF: ultimately fatal, UTI: urinary tract infection, WIC: weighted index of comorbidities.

^a^ intra-abdominal infection, respiratory tract infection, and unknown source.

^b^ Ertapenem nonsusceptible was an ertapenem MIC of>0.25 g/ml, according to the breakpoint criteria of CLSI (document M100-S21).

^c^ Number of days from admission to ESBL culture.

^d^ Time interval from the diagnosis of bacteremia to administration of appropriate antibiotics.

^e^ Others included respiratory tract, skin and soft tissue, and central nervous system infections.

^f^ During the 30 days preceding BSI onset.

The meta-analysis showed that prior antimicrobial therapy (total number of patients, 614; pooled RR, 2.89; 95% CI, 1.22–6.85; *p* = 0.016), neutropenia (total number of patients, 464; pooled RR, 5.58; 95% CI, 2.03–15.35; *p* = 0.0009), nosocomial infection (total number of patients, 1420; pooled RR, 2.46; 95% CI, 1.22–4.95; *p* = 0.012), rapidly fatal underlying disease (total number of patients, 661; pooled RR, 4.21; 95% CI, 2.19–8.08; *p* < 0.0001), respiratory tract infection (total number of patients, 1817; pooled RR, 2.12; 95% CI, 1.33–3.36; *p* = 0.0015), urinary tract infection (total number of patients, 611; pooled RR, 0.15; 95% CI, 0.04–0.57; *p* = 0.0056), PBS (per1) (total number of patients, 688; pooled RR, 1.35; 95% CI, 1.18–1.53; *p* < 0.0001), PBS ≥ 4 (total number of patients, 1534; pooled RR, 4.02; 95% CI, 2.77–5.85; *p* < 0.0001), severe sepsis (total number of patients, 475; pooled RR, 11.74; 95% CI, 4.68–29.43; *p* < 0.0001), severe sepsis or septic shock (total number of patients, 1235; pooled RR, 4.19; 95% CI, 2.83–6.18; *p* < 0.0001), and appropriate empirical therapy (total number of patients, 412; pooled RR, 0.39; 95% CI, 0.18–0.82; *p* = 0.013) were mortality predictors (Figure 2). Moreover, most of these factors had *I*^2^ values < 50% in terms of heterogeneity (prior antimicrobial therapy: *I*^2^ = 28%, Q = 2.77, *p* = 0.25; neutropenia: *I*^2^ = 0%, Q = 1.07, *p* = 0.59; nosocomial infection: *I*^2^ = 57%, Q = 11.51, *p* = 0.04; rapidly fatal underlying disease: *I*^2^ = 16%, Q = 2.37, *p* = 0.31; respiratory tract infection: *I*^2^ = 43%, Q = 12.32, *p* = 0.09; urinary tract infection: *I*^2^ = 42%, Q = 5.21, *p* = 0.16; PBS (per1): *I*^2^ = 38%, Q = 3.22, *p* = 0.20; PBS ≥ 4: *I*^2^ = 10%, Q = 4.44, *p* = 0.35; severe sepsis: *I*^2^ = 45%, Q = 3.62, *p* = 0.16; severe sepsis or septic shock: *I*^2^ = 0%, Q = 0.70, *p* = 0.87; appropriate empirical therapy: *I*^2^ = 19%, Q = 3.72, *p* = 0.29). The funnel plot showed no publication bias for any of the predictive factors. Meanwhile, no statistically significant pooled RR for mortality was detected for the Charlson score (pooled RR, 1.07; 95% CI, 0.94–1.21; *p* = 0.30) and piperacillin/tazobactam (pooled RR, 1.80; 95% CI, 0.81–3.98; *p* = 0.15). Moreover, both these factors had *I*^2^ values >50% in terms of heterogeneity (Charlson score, 51%; piperacillin/tazobactam, 55%). Figure 2.Forest plot of risk ratio for mortality of patients with (a) prior antimicrobial therapy, (b) neutropenia, (c) nosocomial infection, (d) rapidly fatal underlying disease, (e) respiratory tract infection, (f) urinary tract infection, (g) Pitt bacteremia score (per1), (h) Pitt bacteremia score ≥4, (i) severe sepsis, (j) severe sepsis or septic shock, and (k) appropriate empirical therapy for extended-spectrum beta-lactamase-producing Enterobacteriaceae bacteremia.

**Figure:**
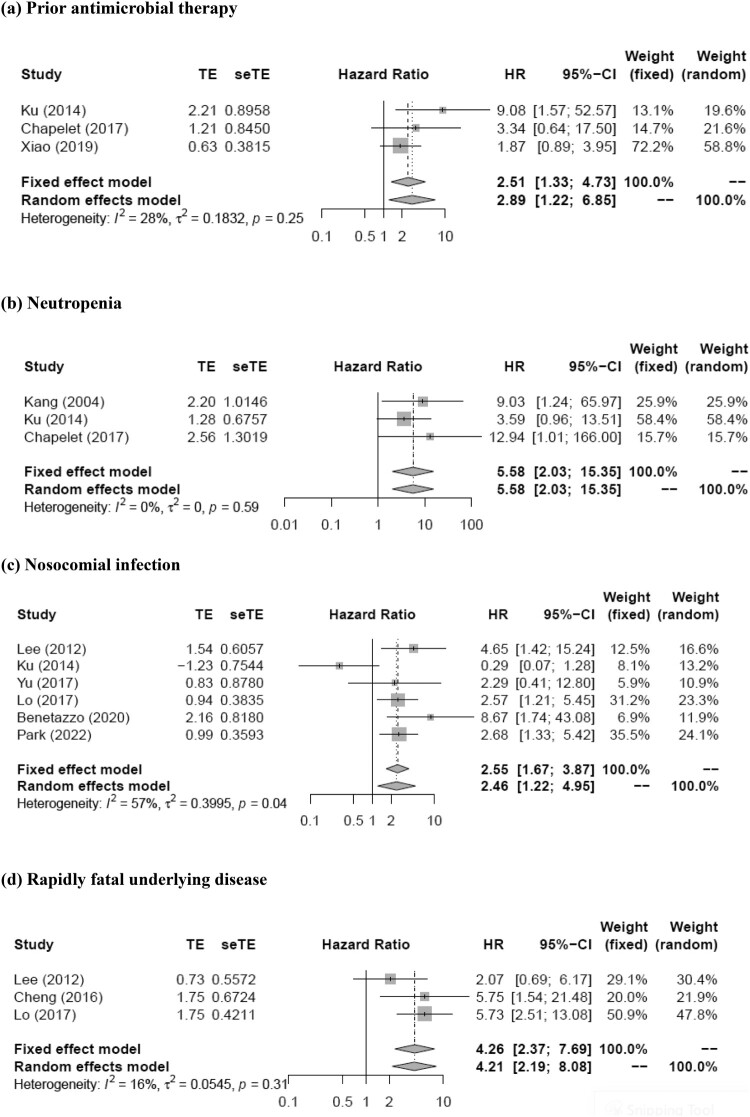

**Figure:**
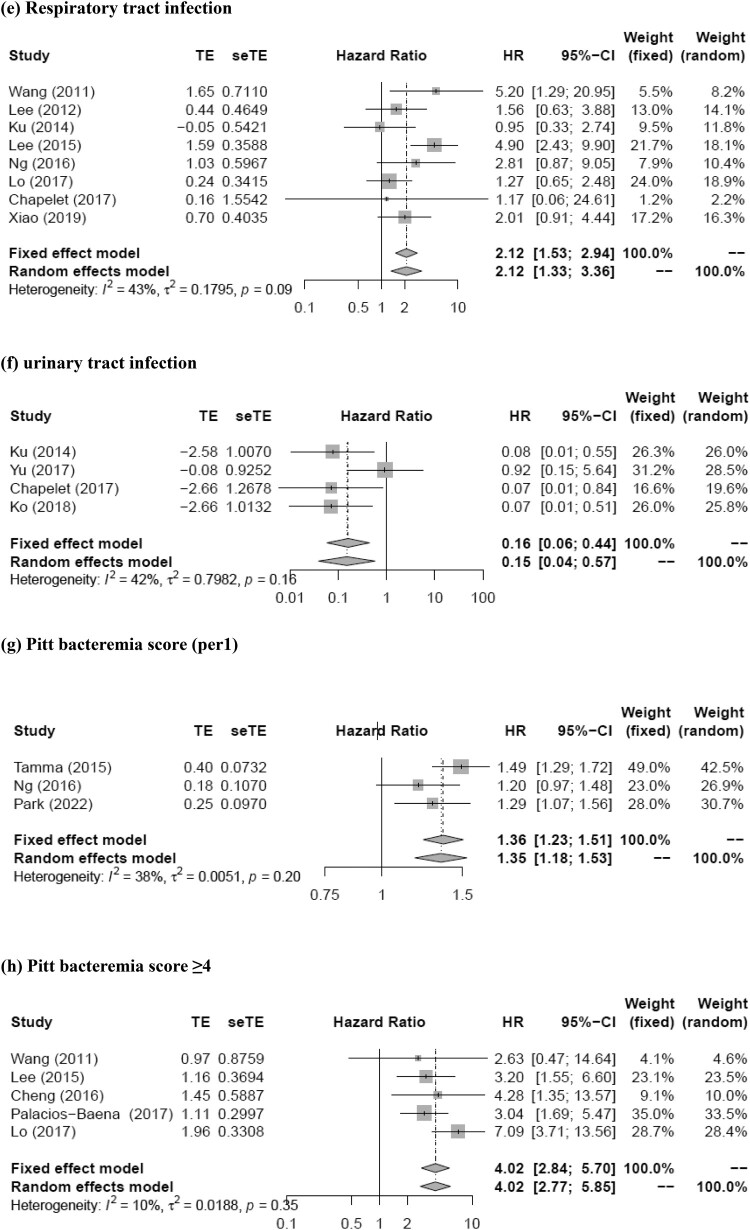

**Figure:**
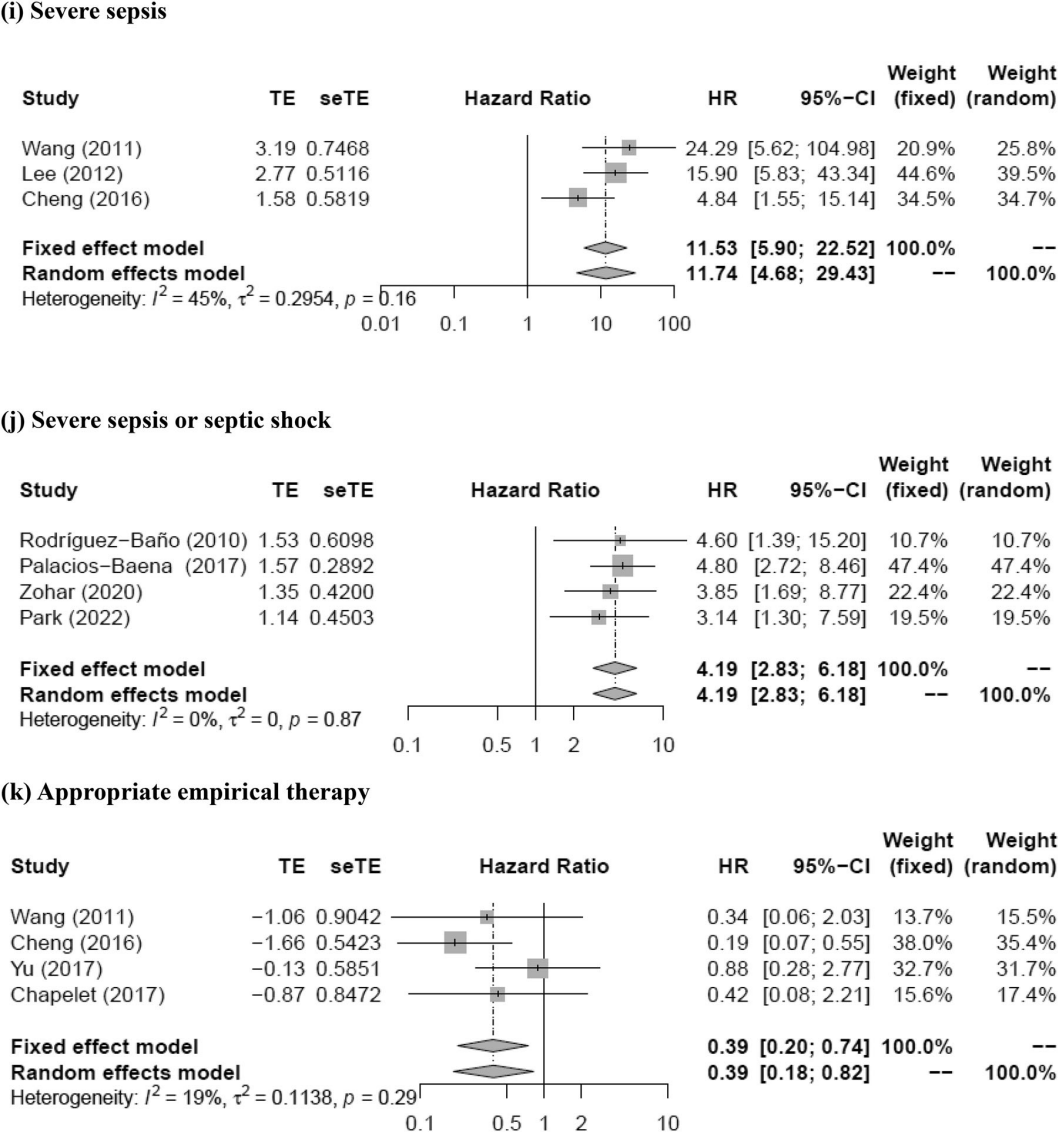

**Figure 3.**
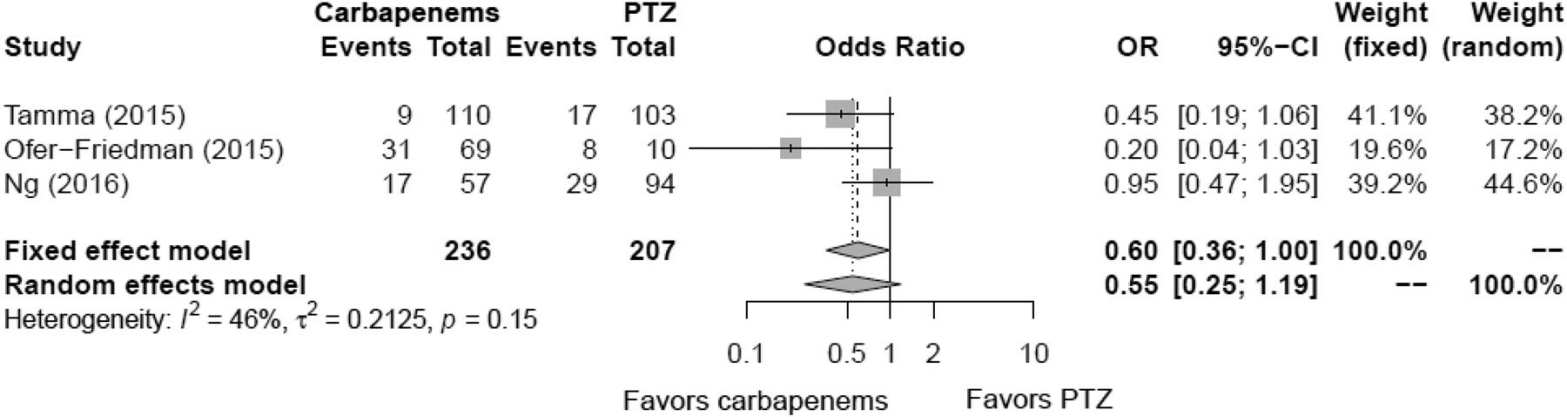
Forest plot showing the odds ratio of the mortality for carbapenems *versus* non-carbapenems in patients with extended-spectrum beta-lactamase-producing Enterobacteriaceae bacteremia.

Using subgroup analysis, the mortality associated with ESBL-PE bacteremia based on carbapenem- and carbapenem-sparing regimens is summarized in Table 3. Identical regimens were reported in three or more studies and included only carbapenems *versus* piperacillin-tazobactam (PTZ). Data from three studies, involving 443 patients, were subjected to meta-analysis of mortality rate. Carbapenems and PTZ were administered to 236 and 207 patients, respectively. Mortality occurred in 57 patients (24.2%) receiving carbapenems and 54 patients (26.1%) receiving PTZ. The meta-analysis showed no statistically significant difference in mortality among the two groups (Favours carbapenems: pooled RR, 0.55; 95% CI, 0.25–1.19; *p* = 0.13) (Figure 3). Moreover, this factor had *I*^2^ values of < 50% in terms of heterogeneity (*I*^2^ = 46%, Q = 3.73, *p* = 0.15).

**Table 3.** Summary of mortality in ESBL-PE bacteremia patients according to antibiotic comparisons.

Author (year)	Antibiotic comparison	no. of patients, n/N (%)
Kang et al. [17]	carbapenems vs ciprofloxacin (definitive)	8/62 (12.9) vs 3/29 (10.3)
Tamma et al. [27]	carbapenems vs PTZ (empirical)	9/110 (8.2) vs 17/103 (16.5)
Ofer-Friedman et al. [28]	carbapenems vs PTZ (empirical or definitive)	31/69 (44.9) vs 8/10 (80.0)
Lee et al. [29]	carbapenems vs flomoxef (definitive)	33/257 (12.8) vs 38/132 (28.8)
Ng et al. [30]	carbapenems vs PTZ (empirical)	17/57 (29.8) vs 29/94 (30.9)
Lo et al. [32]	carbapenems vs fluoroquinolone (definitive)	64/275 (23.3) vs 2/24 (8.3)
Ko et al. [13]	carbapenems vs non-carbapenems (empirical)	20/175 (11.4) vs 3/48 (6.3)
Xiao et al. [19]	carbapenems vs BLBLI combination (empirical)	15/117 (12.8) vs 17/95 (17.9)

Notes: BLBLI: beta-lactam-beta-lactamase inhibitor, n: sample number, N: sample size, no: numero sign, PTZ: piperacillin-tazobactam, vs: versus.

## Discussion

To date, studies investigating the predictors of mortality in patients with ESBL-PE bacteremia have yielded inconsistent results. Therefore, access to a systematic and comprehensive summary of the existing evidence is essential for all clinicians involved in the care of patients with infectious diseases to ensure appropriate diagnosis, treatment, and preventive measures. In this systematic literature review, we assessed 22 observational studies on ESBL-PE bacteremia published between 2004 and 2022, which included 4607 patients, approximately 7.5 times the number of patients included in the largest simplex research. The meta-analysis revealed that prior antimicrobial therapy, neutropenia, nosocomial infection, rapidly fatal underlying disease, respiratory tract infection, urinary tract infection, PBS (per1), PBS ≥ 4, severe sepsis (or septic shock), and appropriate empirical therapy were predictors of mortality caused by ESBL-PE bacteremia. Of the above predictors, nosocomial infection, respiratory tract infection, and appropriate empirical therapy are considered to be of particular clinical importance.

In addition to the articles included in this review, a few studies reported that nosocomial infection was a predictor of mortality from ESBL-PE bacteremia [35,36]. A recent study also showed that nosocomial ESBL-PE bacteremia are associated with higher mortality compared with community-onset ESBL-PE bacteremia [18]. We believe that two factors play a major role in the high mortality associated with nosocomial ESBL-PE bacteremia: differences in pathogenicity and differences in antimicrobial susceptibility. Regarding differences in pathogenicity, some studies reported that the frequency of highly pathogenic sequence type 131 C1/H30-R and/or C2/H30-Rx in nosocomial ESBL *E. coli* was relatively high [37,38]. Furthermore, Liu et al. showed that the incidence of hypervirulent strains in nosocomial ESBL *K. pneumoniae* has increased [39]. Meanwhile, regarding differences in antimicrobial susceptibility, previous studies indicated that nosocomial ESBL-producing isolates were more resistant than community-acquired isolates [40,41]. Therefore, patients with nosocomial infection may receive inappropriate antimicrobial therapy more frequently than community-acquired patients. A few studies have reported that the length of hospital stay from admission to onset is longer in non-survivors of ESBL-PE bacteremia than in the survivors [19,42]. A prolonged hospital stay can lead to adverse events, including nosocomial infections and a decline in functional status [43]. Furthermore, Marfil-Garza et al. demonstrated that a prolonged hospital stay is associated with increased mortality and other poor outcomes [44]. The abovementioned factors support the finding that nosocomial infection is associated with an increased risk mortality in patients with ESBL-PE bacteremia.

In addition to the articles examined in our review, a few studies have cited respiratory tract infection as a predictor of mortality caused by ESBL-PE bacteremia [6,45]. Cheng et al. reported that both severity and mortality among patients with bacteremic pneumonia caused by ESBL-producing *E. coli* or *K. pneumoniae* were very high (PBS ≥4, 42.3%; severe sepsis, 52.3%; crude mortality, 55.9%) [20]. Furthermore, Harada et al. showed that higher amounts of exposed bacteria in pulmonary infections caused by ESBL *K. pneumoniae* increased the minimal inhibitory concentration of antibiotics due to the inoculum effect [46]. These findings support that respiratory tract infection is associated with an increased risk of mortality in patients with ESBL-PE bacteremia. Therefore, we need to be particularly vigilant for respiratory tract infections among various infection sources in patients with ESBL-PE bacteremia.

In addition to the articles included in this review, a few studies have reported that appropriate empirical therapy was a protective factor against mortality from ESBL-PE bacteremia [47]. Biehl et al. indicated that inadequate treatment for patients with ESBL-PE infection led to worse outcomes and survival [48]. Furthermore, Gutiérrez-Gutiérrez and Rodríguez-Baño reported that delay in initiating active antibiotic therapy may be associated with a high mortality rate for ESBL infections [49]. These findings suggest that appropriate empirical therapy is associated with a decreased mortality risk in patients with ESBL-PE bacteremia. In our study, PTZ did not account for a statistically significant increase in mortality compared with carbapenems. However, the number of studies and cases may be too low for definitive conclusions. Current treatment options for ESBL-PE infections include ceftolozane/tazobactam, ceftazidime/avibactam, aminoglycosides, and fosfomycin, as well as carbapenems and PTZ [50]. Selecting appropriate antimicrobial agents for ESBL-PE bacteremia according to infection source and severity is very important. Therefore, to establish the optimal treatment for patients with ESBL-PE bacteremia, we need to collect and analyze data from more patients and compare the clinical effectiveness of carbapenems and carbapenem-sparing regimens.

In addition to the articles included in this review, a few studies have reported that prior antimicrobial therapy was a predictor of mortality from ESBL-PE bacteremia [51,52]. However, in general, prior antimicrobial therapy leads to the acquisition of or infection by multi-drug resistant organisms, instead of mortality [53]. Ku et al. reported that the association between prior antimicrobial therapy and increased mortality might be due to a high degree of antimicrobial resistance of causative organisms isolated from patients with a history of antimicrobial therapy [21]. Therefore, such patients may develop more complicated infections with a poor prognosis. These findings suggest that whether prior antimicrobial therapy is a predictor of mortality from ESBL-PE bacteremia is debatable. Future studies are required to collect and review further data related to prior antimicrobial therapy and increased mortality.

The present study has several limitations. First, we used only two databases (PubMed and Cochrane Library) for the literature search. Second, we limited our selection to only articles written in English, which restricts the scope of our analysis. Third, although we attempted to minimize the effects of confounding factors as much as possible by excluding studies with inappropriate multivariate analysis and by performing a meta-analysis using a random-effect model, we were unable to completely eliminate their influence. Fourth, most studies used a retrospective study design and may have been susceptible to selection bias. Fifth, the outcome of mortality has not been assessed at the same point of time in all studies; nevertheless, it was assessed at 30 days in approximately half of the studies. Sixth, obtaining a clear inference from the current evidence may be difficult because some studies may have included participants with polymicrobial bacteremia or resistance genes other than ESBL. Seventh, concerns remain about the sample size for each predictor as only three to five studies were included in most meta-analyses. Finally, we performed the analysis without unifying the bacterial species or considering the genotypes. Therefore, future studies are required to analyze the bacterial species or genotypes in ESBL-PE causing bacteremia during data collection and to further establish the predictive factors against mortality.

In conclusion, the present study demonstrated that prior antimicrobial therapy, neutropenia, nosocomial infection, rapidly fatal underlying disease, respiratory tract infection, urinary tract infection, PBS (per1), PBS ≥ 4, severe sepsis (or septic shock), and appropriate empirical therapy are predictors of mortality caused by ESBL-PE bacteremia. Therefore, patients with ESBL-PE bacteremia who have the aforementioned factors require prudent management for improved outcomes. Based on the above findings, we believe that this research will lead to better treatment and improvement of the clinical outcomes of patients with ESBL-PE bacteremia.

## Data Availability

All data generated or analyzed during this study are included in this published article.
